# Draft Genome Sequence of the Pediocin-Encoding Biopreservative and Biocontrol Strain *Pediococcus acidilactici* D3

**DOI:** 10.1128/genomeA.00208-13

**Published:** 2013-06-20

**Authors:** Joseph M. Sturino, Mahitha Rajendran, Eric Altermann

**Affiliations:** Nutrition and Food Science Department, Texas A&M University, College Station, Texas, USAa; Intercollegiate Genetics Program, Texas A&M University, College Station, Texas, USAb; AgResearch Ltd., Palmerston North, New Zealandc; Riddet Institute, Massey University, Palmerston North, New Zealandd

## Abstract

We describe a draft genome sequence for *Pediococcus acidilactici* strain D3, a component of multistrain commercial cultures with biopreservative and biocontrol properties in food-based applications. Strain D3 encodes at least one antimicrobial peptide, pediocin AMPd3. The AMPd3-encoding operon exhibits high sequence similarity to the archetype pediocin, PA-1, encoded by *P. acidilactici* PAC 1.0.

## GENOME ANNOUNCEMENT

Approximately 1.3 billion tons of edible food is either lost or wasted annually, which represents nearly one-third of the global food mass that is produced for human consumption each year (1, 2). As the world population is expected to grow by more than 2.3 billion people between 2009 and 2050, the reduction of food loss and waste is a critical benchmark to improve food security and environmental sustainability across the globe (3). Innovative technologies, including biopreservative and biocontrol cultures, are being developed to address these grand challenges.

Biopreservative and biocontrol cultures are living microorganisms that, when applied in adequate amounts, extend the storage life and safety, respectively, of beverages, foods, or feeds without changing their organoleptic properties (4, 5). *Pediococcus acidilactici* D3 is a component of lactic acid bacterium (LAB)-based biocontrol cultures that are generally recognized as safe (GRAS) for use in meat products (6). The antimicrobial functionality of these cultures (7, 8, 9) is likely due, in part, to the production of bacteriocins and low-molecular-weight metabolites, including lactic acid (10, 11); however, strain-specific molecular and generic evidences to confirm these suppositions are lacking. As a result, a draft sequence of the D3 genome was generated and examined for genes that might explain its antimicrobial qualities.

To this end, a paired-end library of D3 genomic DNA was prepared and sequenced using the Genome Analyzer IIx (Illumina, San Diego, CA) by the Texas AgriLife Genomics and Bioinformatics Center (College Station, TX). The size of the library inserts ranged from 197 to 557 nucleotides (mean, 305 nucleotides). Based on the size of the *P. acidilactici* DSMZ 20284^T^ reference genome (1.93 Mb), a sequencing coverage of approximately 2,289-fold was inferred. The reads were assembled *de novo* with SPAdes version 2.2.1 (12). In total, 512 contigs with lengths of >1,000 bp were obtained.

Based upon alignments with the *P. acidilactici* neotype strain, DSMZ 20284^T^, a D3 pseudochromosome was generated using the FGD and ACT software packages (13, 14). The size (1,962,090 Mb) and G+C content (42.09%) of the D3 draft genome were comparable to those of other previously sequenced pediococci, including *P. acidilactici* MA18/5M (1.99 Mb and 42% G+C) (15) (GenBank accession no. AGKB00000000.1), *P. acidilactici* DSMZ 20284^T^ (1.93 Mb and 42% G+C) (accession no. NZ_AEEG00000000.1), *P. acidilactici* 7_4 (2.01 Mb and 42% G+C) (accession no. NZ_ACXB00000000.1), and *Pediococcus pentosaceus* ATCC 25745 (1.83 Mb and 37.4% G+C) (accession no. CP000422).

Global Annotation of Mulitplexed On-site bLasted DNA-sequences (GAMOLA) version 2 was used to predict protein-coding domain sequences and to annotate the draft genome (16). Based on these analyses, 1,976 protein-coding genes were predicted. Unlike strains DSMZ 20284^T^, 7_4, and MA18/5M, D3 carries four genes (*pedABCD*) tentatively related to the immunity to and expression of an antimicrobial peptide (pediocin), designated AMPd3. The AMPd3-deduced protein sequence is identical to that of an archetype pediocin, PA-1, encoded by *P. acidilactici* PAC 1.0 (17).

### Nucleotide sequence accession numbers.

The Whole-Genome Shotgun project was deposited at DDBJ/EMBL/GenBank under the accession no. AQGT00000000. The version described in this paper is the first version, accession no. AQGT01000000.
